# A modified variable flip angle release device for endoscopic titanium clips

**DOI:** 10.1055/a-2584-1271

**Published:** 2025-05-09

**Authors:** Jiaxing Feng, Chunping Zhang, Jiachen Bao, Qingyu Xu, Yutong Gan, Yiqun Hu

**Affiliations:** 1 66366 Department of Gastroenterology, The National Key Clinical Specialty, Zhongshan Hospital of Xiamen University, School of Medicine, Xiamen University, Xiamen, China; 2 66366 Clinical Research Center for Gut Microbiota and Digestive Diseases of Fujian Province, Xiamen Key Laboratory of Intestinal Microbiome and Human Health, Xiamen, China; 3 Institute for Microbial Ecology, Department of Digestive Disease, School of Medicine, Xiamen University, Xiamen, China

Titanium clips are widely used in gastrointestinal endoscopy, especially during endoscopic surgical procedures. Their principal role is to efficiently close defects within the gastrointestinal tract, thereby mitigating the risk of postoperative complications 1 2 3 4 . During clinical procedures, the alignment of the endoscope with the gastrointestinal lumen, coupled with the ongoing peristaltic movements of the stomach and intestinal cavities, frequently leads to suboptimal angulation for conventional titanium clip applicators when deploying clips via the endoscopeʼs channel, impeding the precise release of clips. Furthermore, the restricted opening angle of traditional titanium clips complicates their accurate placement on the wound surface, presenting considerable operational challenges.

Considering this, we have developed an innovative adjustable-angle endoscopic titanium clip release device. This device is composed of a clamp head assembly, a spring tube, a handle assembly, and an angle adjustment mechanism ( Fig. 1 ). The clamp head assembly is welded to the spring tube, while the angle adjustment mechanism incorporates a side-pulling steel wire and an end pull ring, which is also welded to the tail of the titanium clip. In its static configuration, the side-pulling steel wire remains linear. Upon activation of the pull ring at the terminus of the angle adjustment mechanism, the side-pulling steel wire contracts, reducing the distance between the titanium clip and the junction of the spring tube and handle assembly, thereby bending the titanium clip to the desired angle ( Fig. 2 ). The titanium clip is comprised of a tail end and a clip section. The tail end provides leverage during deployment to facilitate smooth clamping. The fully extended length of the titanium clip is approximately 10–15 mm, with an opening angle range of 90–135° ( Video 1 ).

**Fig. 1:**
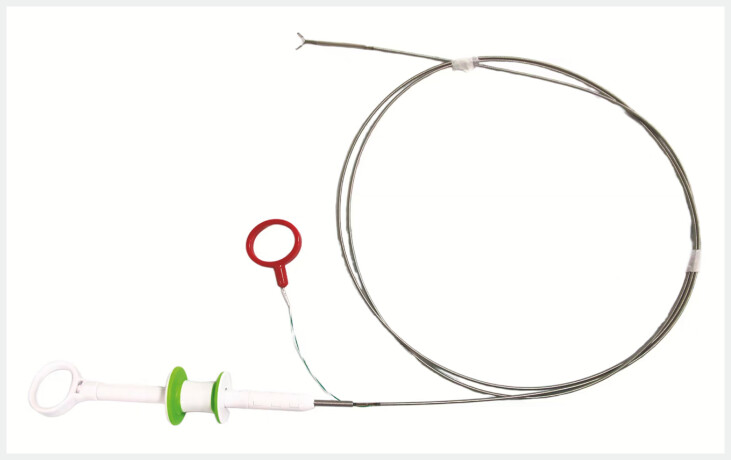
The modified release device requires a clamp head assembly, a spring tube, a handle assembly, and an angle adjustment mechanism.

**Fig. 2:**
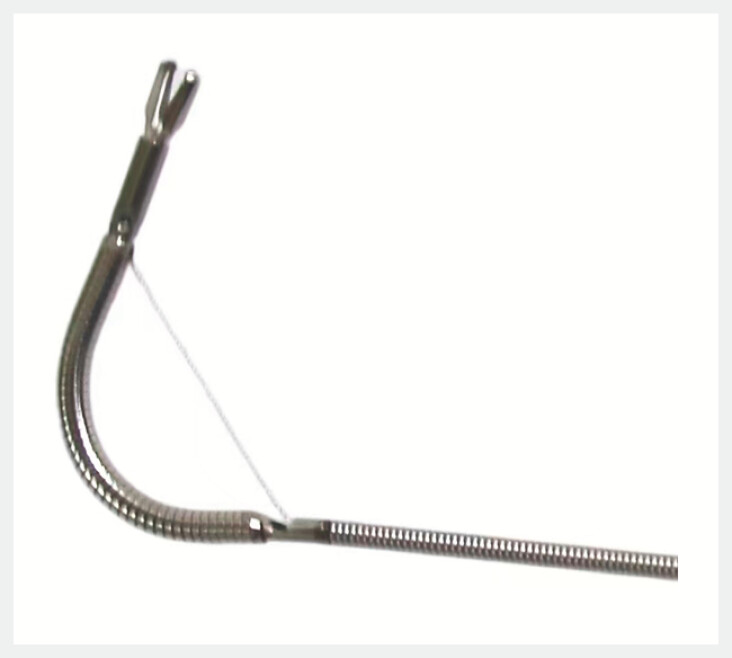
Pulling the ring tightens the wire, bending the clip to the desired angle.

A modified variable flip angle release device for endoscopic titanium clips.Video 1

In summary, this modified variable-angle titanium clip-release device adapts to wounds throughout the digestive tract for more precise treatment.

Endoscopy_UCTN_Code_TTT_1AO_2AD

## Notes

Endoscopy E-Videos https://eref.thieme.de/e-videos
*E-Videos* is an open access online section of the journal *Endoscopy* , reporting on interesting cases and new techniques in gastroenterological endoscopy. All papers include a high-quality video and are published with a Creative Commons CC-BY license. Endoscopy E-Videos qualify for HINARI discounts and waivers and eligibility is automatically checked during the submission process. We grant 100% waivers to articles whose corresponding authors are based in Group A countries and 50% waivers to those who are based in Group B countries as classified by Research4Life (see: https://www.research4life.org/access/eligibility/ ). This section has its own submission website at https://mc.manuscriptcentral.com/e-videos .

## References

[1] Qin G Wang QQ Tan C A novel method for efficient closure of large mucosal defects using nylon loops combined with titanium clips after endoscopic submucosal dissection Endoscopy 2023 55 01 E848 E849 37369242 10.1055/a-2106-2383 PMC10299868

[2] Mou H Liu Q Fan Y Nylon ring with titanium clip assists endoscopic cyanoacrylate injection for the treatment of GOV1-type gastric varices Endoscopy 2023 55 01 E578 E580 37011901 10.1055/a-2011-5595 PMC10070010

[3] Yu J Zhou CJ Wang P Endoscopic titanium clip closure of gastric fistula after splenectomy: A case report World J Clin Cases 2018 6 1047 1052 10.12998/wjcc.v6.i15.1047 30568962 PMC6288501

[4] Zhan X Wang B Di D Endoscopic closure of gastric tube perforations with titanium clips: a four-case report World J Surg Oncol 2015 13 25 25889662 10.1186/s12957-015-0434-8 PMC4336678

